# Supplementary material for: Prior exposure to B. pertussis shapes the mucosal antibody response to acellular pertussis booster vaccination
Source: Nat Commun. 2022 Dec 2;13:7429. doi: 10.1038/s41467-022-35165-w (PMC9716536; doi:10.1038/s41467-022-35165-w)
Supplement: Supplementary file 1 — Supplementary Information [file 41467_2022_35165_MOESM1_ESM.pdf]

Figure S1

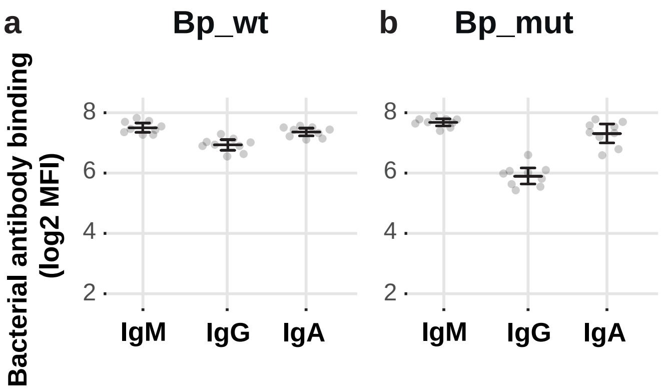
a
Bp_wt
b
Bp_mut
Bacterial antibody binding
(log2 MFI)
8
6
4
2
IgM
IgG
IgA

Figure S1. Variation in antibody binding to *Bp_wt* and *Bp_mut* using positive control samples. *Bp_wt* and *Bp_mut* strains were incubated with heat-inactivated Normal Human Serum (NHS) and antibody binding was subsequently measured by flow cytometry. Log2-transformed mean fluorescence intensity (MFI) of IgM, IgG and IgA for *Bp_wt* **(a)** and *Bp_mut* **(b)**. Experiments were performed independently 9 times. Data is shown as geometric mean with 95% confidence intervals.

Figure S2

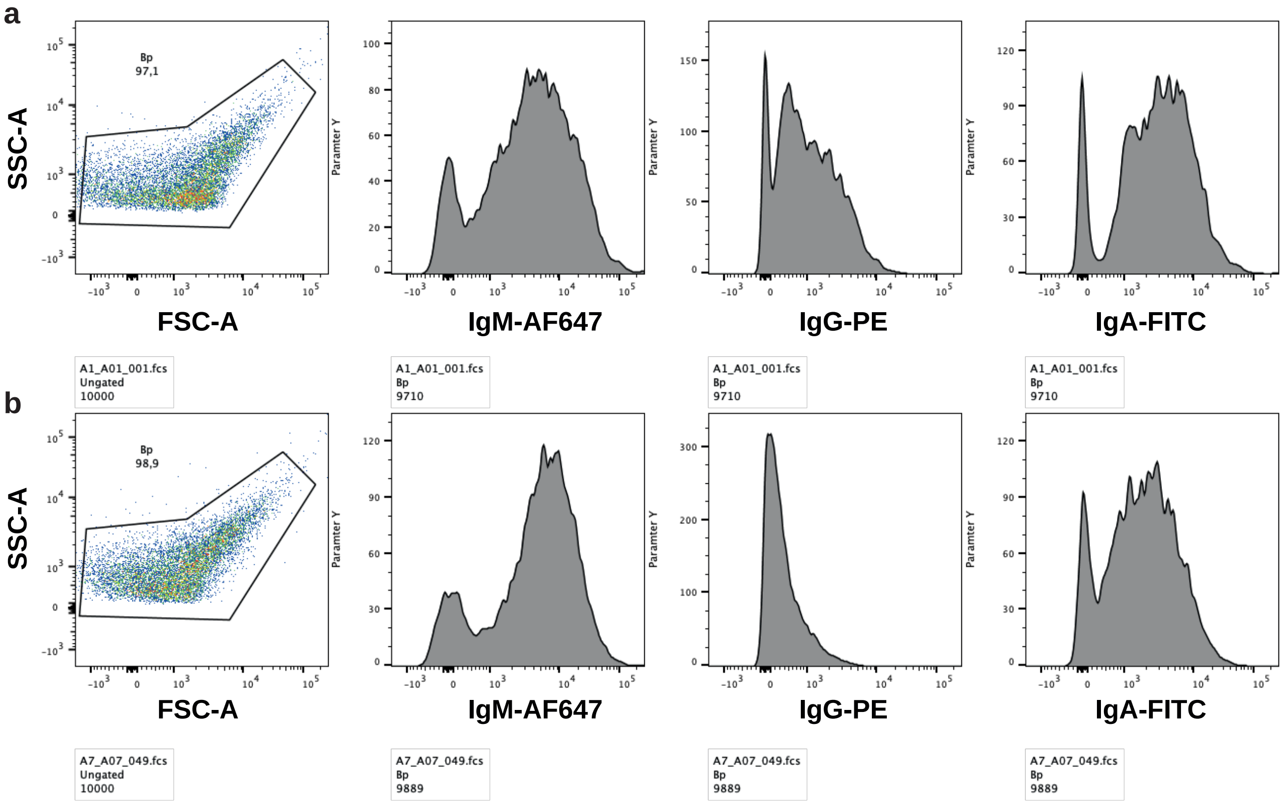
a
b
SSC-A
FSC-A
IgM-AF647
IgG-PE
IgA-FITC
Paramter Y
Bp
97,1
98,9
A1_A01_001.fcs
Ungated
10000
Bp
9710
A7_A07_049.fcs
Ungated
10000
Bp
9889

Figure S2. Gating strategy for IgM, IgG, and IgA mean fluorescence intensities. *Bp_wt* **(a)** and *Bp_mut* **(b)** were first gated on forward scatter (FSC-A)/side scatter (SSC-A). Of this gated population, the mean fluorescence intensities of IgM, IgG, and IgA were determined.

Figure S3

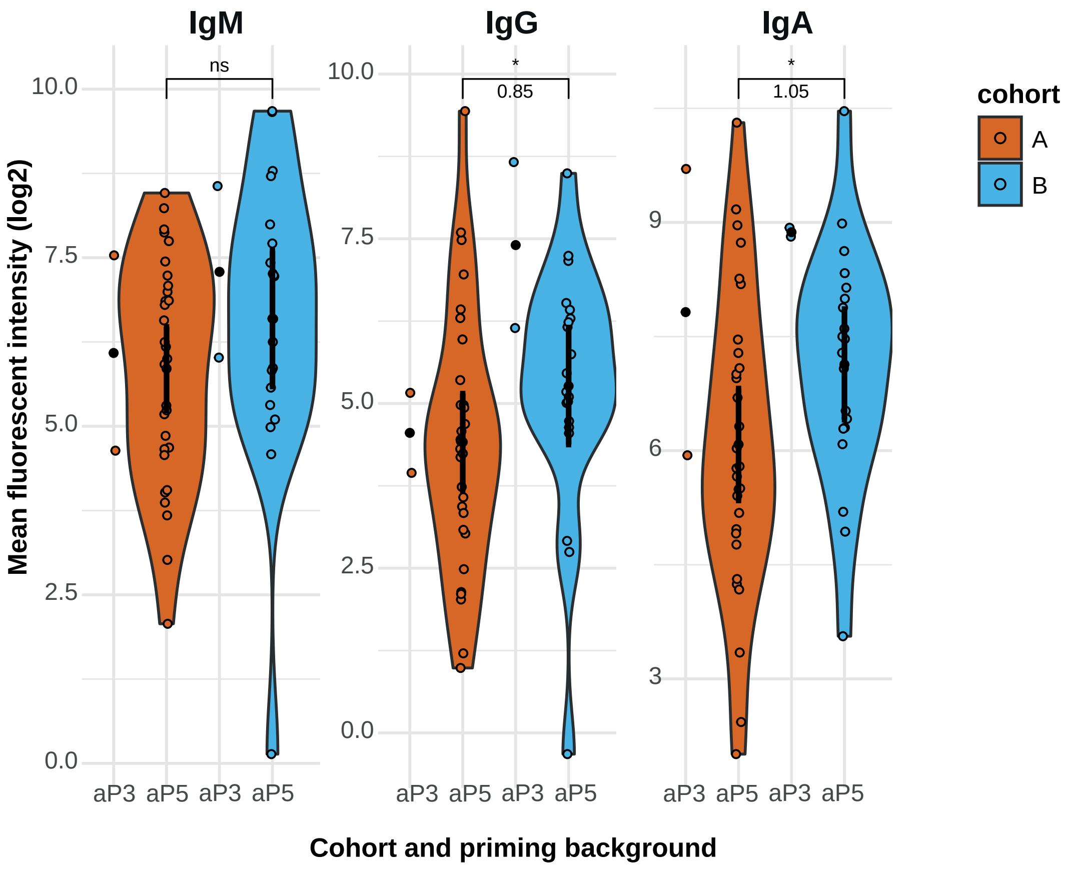

IgM
IgG
IgA
ns
*
0.85
*
1.05
10.0
7.5
5.0
2.5
0.0
9
6
3
Mean fluorescent intensity (log2)
aP3 aP5 aP3 aP5
aP3 aP5 aP3 aP5
aP3 aP5 aP3 aP5
Cohort and priming background
cohort
A
B

Figure S3. Baseline variation in mucosal antibody binding to *Bp_mut*. *Bp_mut*, deficient for FHA, PRN, and PT, was incubated with heat-inactivated MLF and antibody binding to bacteria was subsequently measured by flow cytometry. Log2-transformed mean fluorescence intensity (MFI) of IgM, IgG and IgA binding to *Bp_mut* stratified according to primary vaccination background (aP3 or aP5) per aP-primed cohort (cohort A and cohort B). Data are N = 32 individuals for cohort A and N= 22 individuals for cohort B. Sample means with 95% confidence intervals (solid black point and line) are plotted. No testing was performed on the aP3 groups, due to the low N. Kruskal-Wallis followed by Wilcoxon rank-sum test was used to compare the aP5 levels across cohorts. * $p \leq 0.05$.

Figure S4

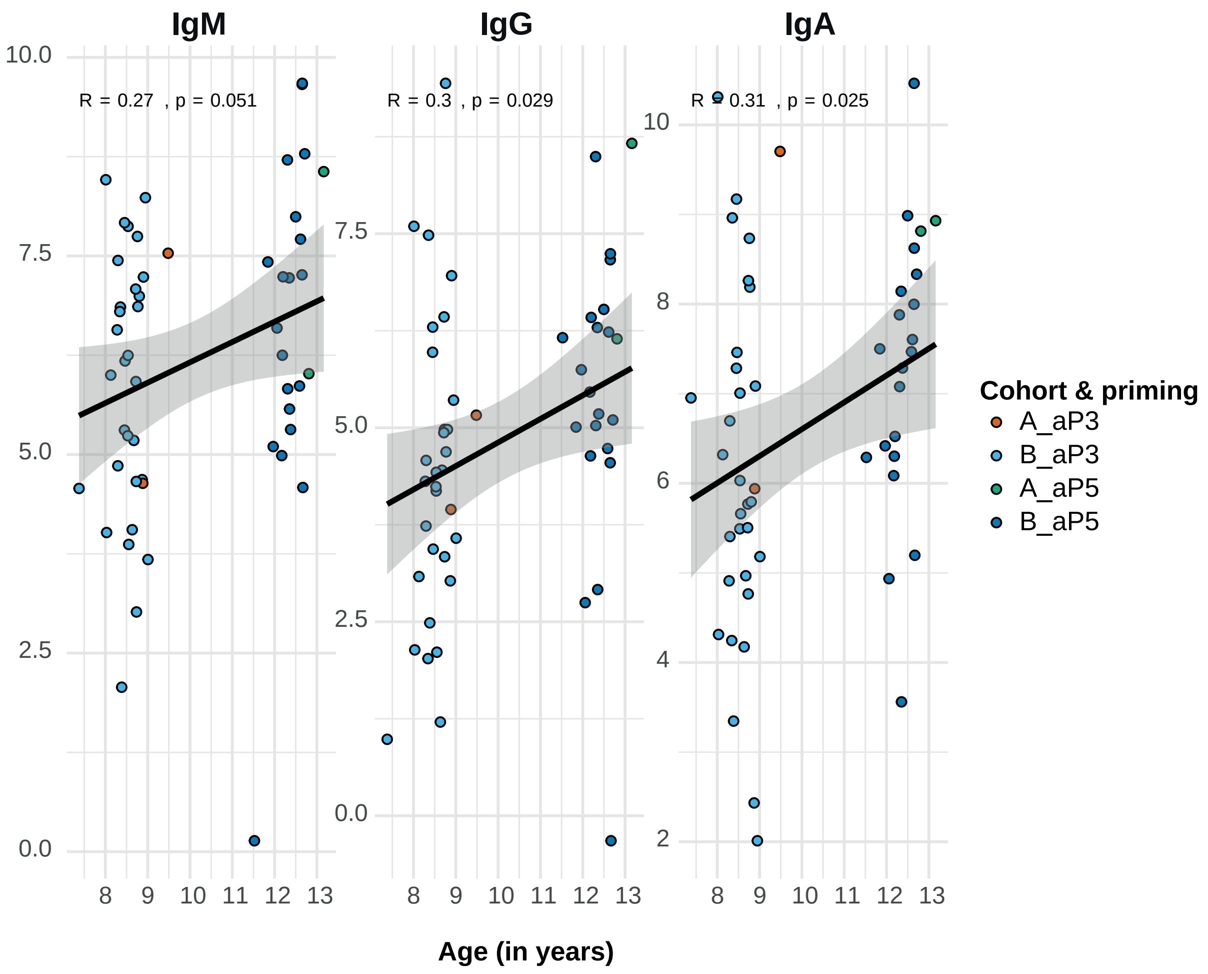
IgM
IgG
IgA
R = 0.27 , p = 0.051
R = 0.3 , p = 0.029
R = 0.31 , p = 0.025
10.0
7.5
5.0
2.5
0.0
10
8
6
4
2
8 9 10 11 12 13
Age (in years)
Cohort & priming
A_aP3
B_aP3
A_aP5
B_aP5

Figure S4. Correlation between baseline *B. pertussis* exposure and age. Pearson correlation of log2-transformed mean fluorescence intensity (MFI) of IgM, IgG, and IgA binding to *Bp_mut* at baseline with the age of participants stratified into priming background (aP3 or aP5) per aP-primed cohort (cohort A and cohort B). Data are N = 2 - 10 per cohort. A two-sided Pearson correlation was performed and the correlation factor R and p-value are depicted in the figure.

Figure S5

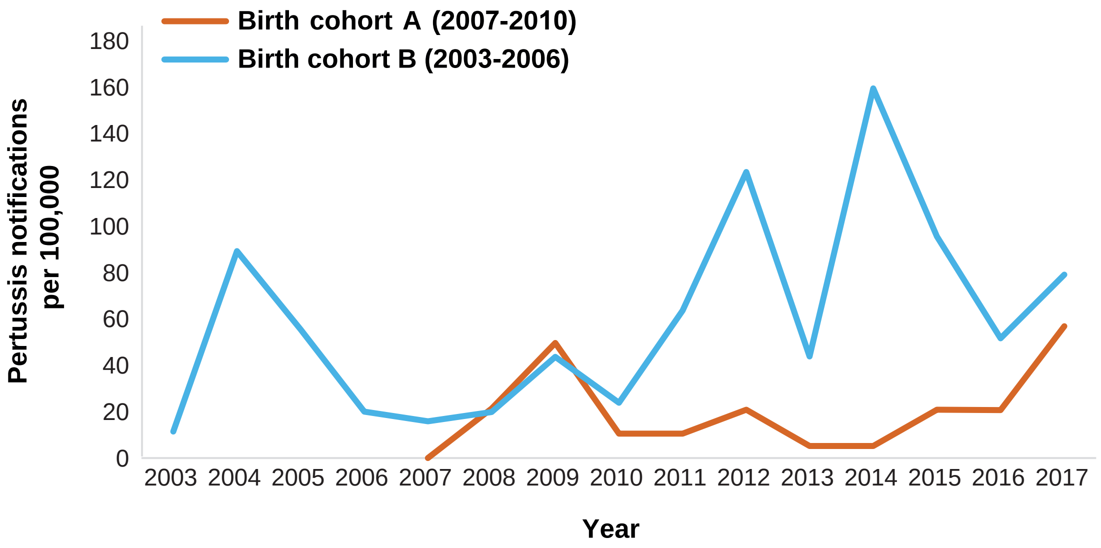

Birth cohort A (2007-2010)
Birth cohort B (2003-2006)
Pertussis notifications per 100,000
180
160
140
120
100
80
60
40
20
0
2003
2004
2005
2006
2007
2008
2009
2010
2011
2012
2013
2014
2015
2016
2017
Year

Figure S5. Annual regional pertussis incidence. Cohorts include children born between 2007-2010 (red line) and between 2003-2006 (blue line), representing the A and B cohorts, respectively. Pertussis disease incidence was obtained for postcode-matched regions where the vaccination study was conducted in the national registry (CBS) and pertussis notifications were obtained from the Dutch National Institute of Health and the Environment (RIVM).

Figure S6

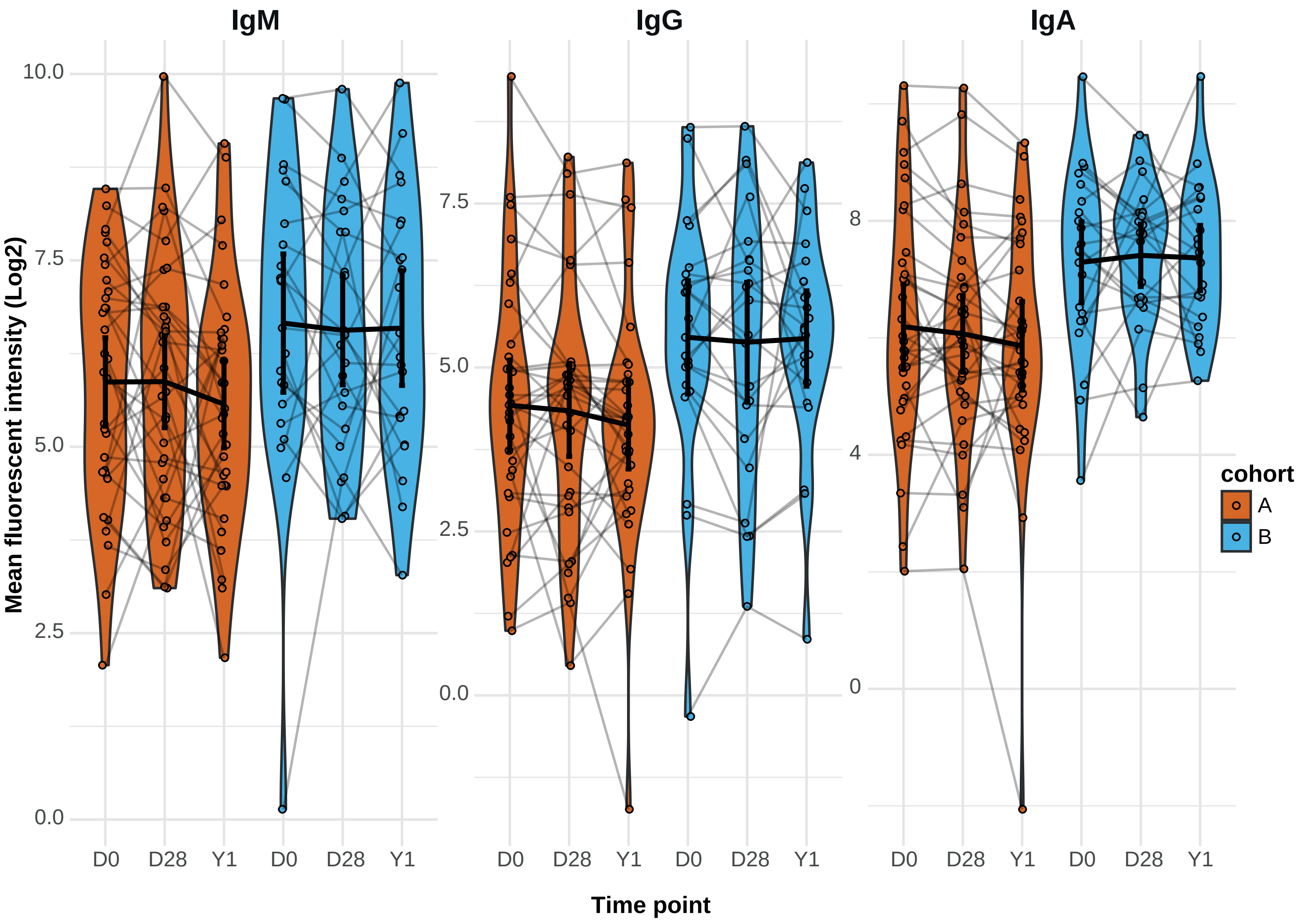

IgM
IgG
IgA
Mean fluorescent intensity (Log2)
10.0
7.5
5.0
2.5
0.0
7.5
5.0
2.5
0.0
8
4
0
D0
D28
Y1
D0
D28
Y1
Time point
cohort
A
B

Figure S6. Vaccination does not increase mucosal antibody binding to *Bp_mut*. MLF was obtained from participants at baseline (D0) and 28 days (D28) and one year (Y1) after a dose of Tdap-IPV. *Bp_mut* was incubated with heat-inactivated MLF and antibody binding to bacteria was subsequently measured by flow cytometry. Log2-transformed mean fluorescence intensity (MFI) of IgM, IgG, and IgA binding to *Bp_mut* is shown. Cohort A and cohort B are indicated by color. Data are N = 32 individuals for cohort A and N= 22 individuals for cohort B. Sample means with 95% confidence intervals (solid black point and line) are plotted.

Figure S7

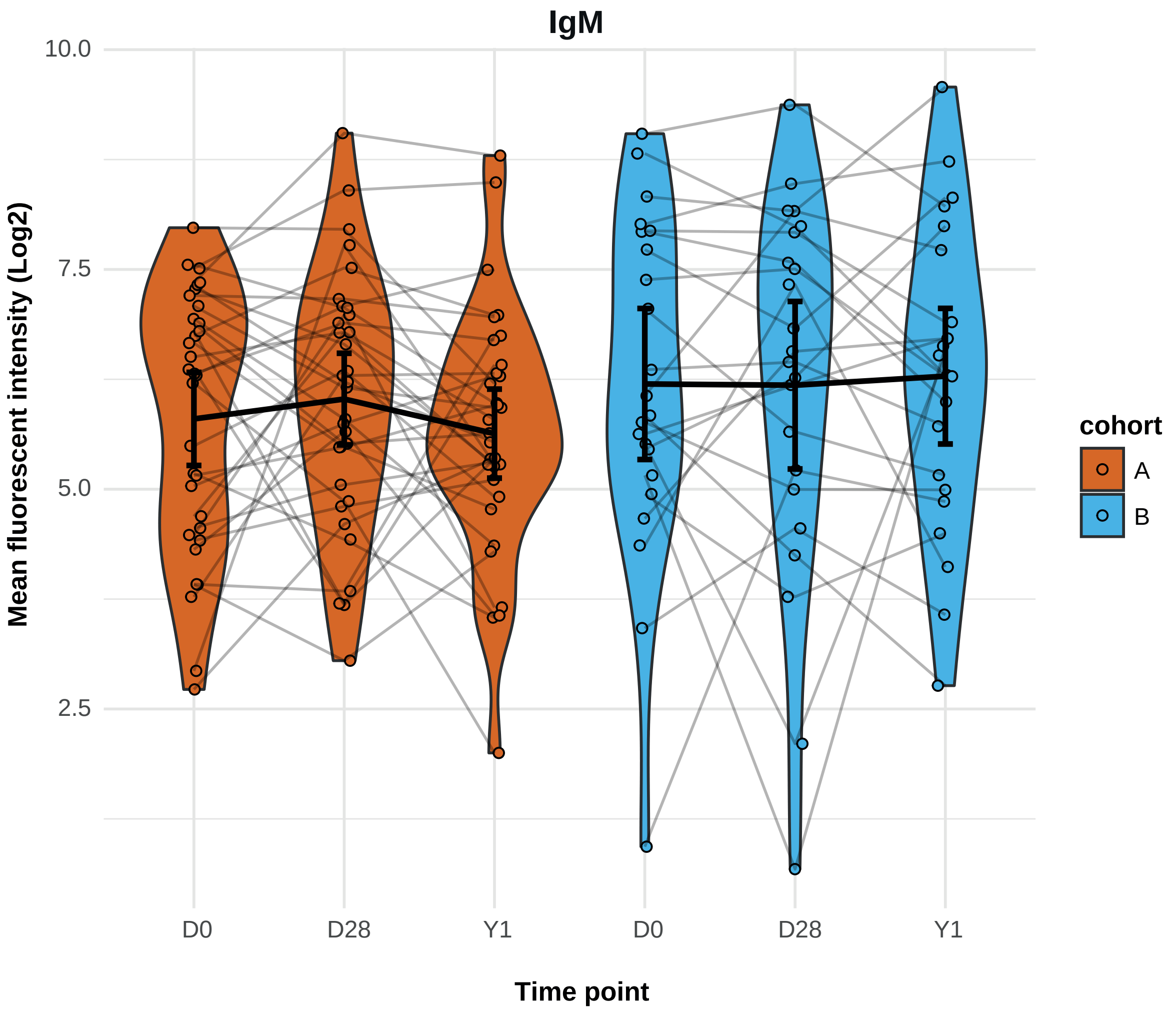
IgM
10.0
7.5
5.0
2.5
Mean fluorescent intensity (Log2)
D0
D28
Y1
D0
D28
Y1
Time point
cohort
A
B

Figure S7. Vaccination does not induce mucosal IgM binding to *Bp_wt*. MLF was obtained from participants at baseline (D0) and 28 days (D28) and one year (Y1) after a dose of Tdap-IPV. *Bp_wt* was incubated with heat-inactivated MLF and antibody binding to bacteria was subsequently measured by flow cytometry. Log2-transformed mean fluorescence intensity (MFI) of IgM to *Bp_wt*. Cohort A and cohort B are indicated by colour. Data are N = 32 individuals for cohort A and N= 22 individuals for cohort B. Sample means with 95% confidence intervals (solid black point and line) are plotted.

Figure S8

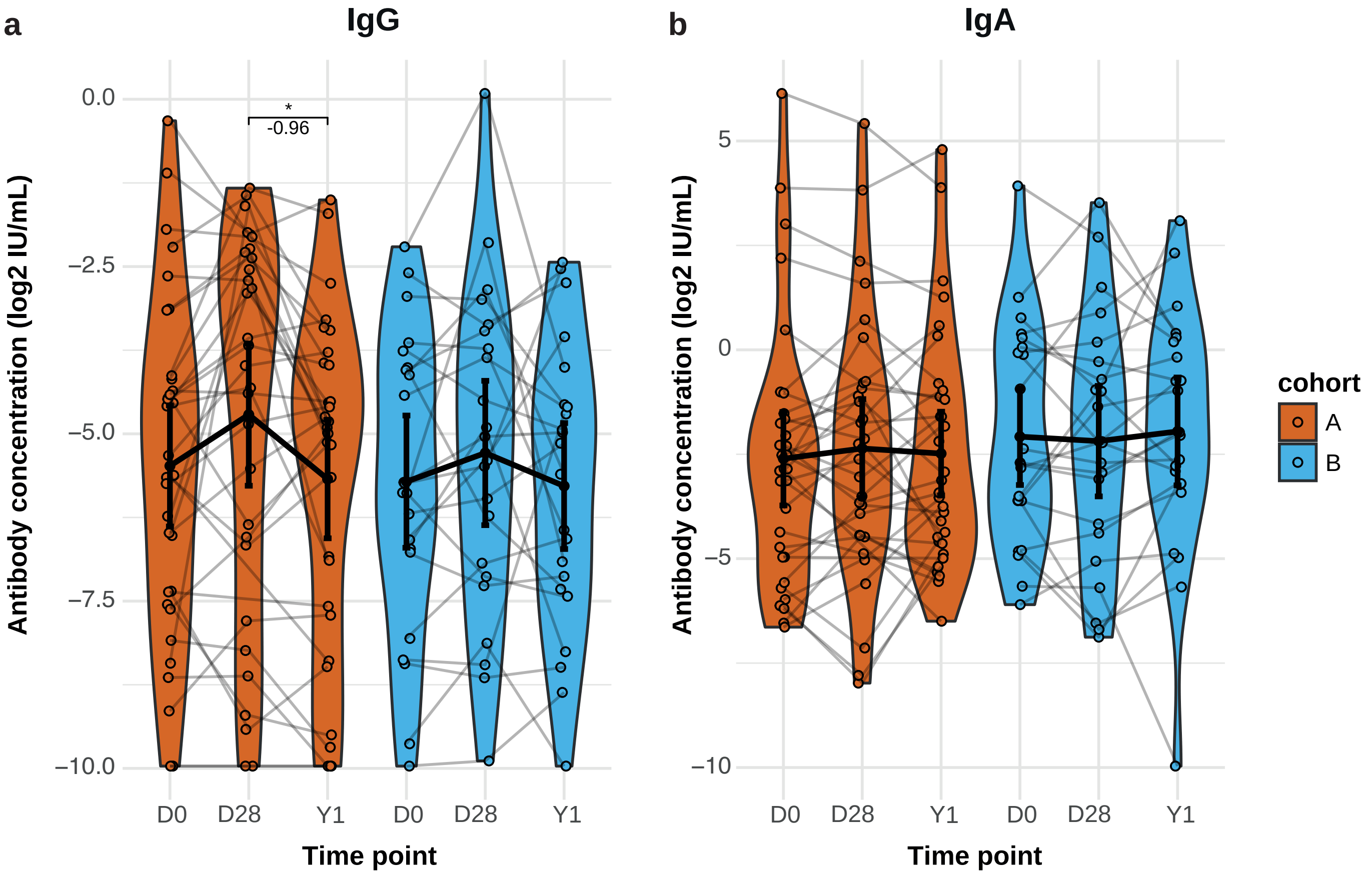
a
IgG
0.0
−2.5
−5.0
−7.5
−10.0
*
-0.96
Antibody concentration (log2 IU/mL)
D0
D28
Y1
D0
D28
Y1
Time point
b
IgA
5
0
−5
−10
Antibody concentration (log2 IU/mL)
D0
D28
Y1
D0
D28
Y1
Time point
cohort
A
B

Figure S8. Effect of vaccination on FIM-specific mucosal antibody concentrations. MLF was obtained from participants at baseline (D0) and 28 days (D28) and one year (Y1) after a dose of Tdap-IPV. Antibody concentrations in MLF were measured by multiplex immunoassay (MIA). Log2-transformed concentrations of FIM2/3-specific IgG (**a**) in international units (AU)/mL and IgA (**b**) in arbitrary units (AU)/mL in MLF at the various sample time points. Data are N = 32 individuals for cohort A and N= 22 individuals for cohort B. Sample means with 95% confidence intervals (solid black point and line) are plotted. Kruskal-Wallis followed by Wilcoxon signed-rank test was used to compare within one cohort

Figure S9

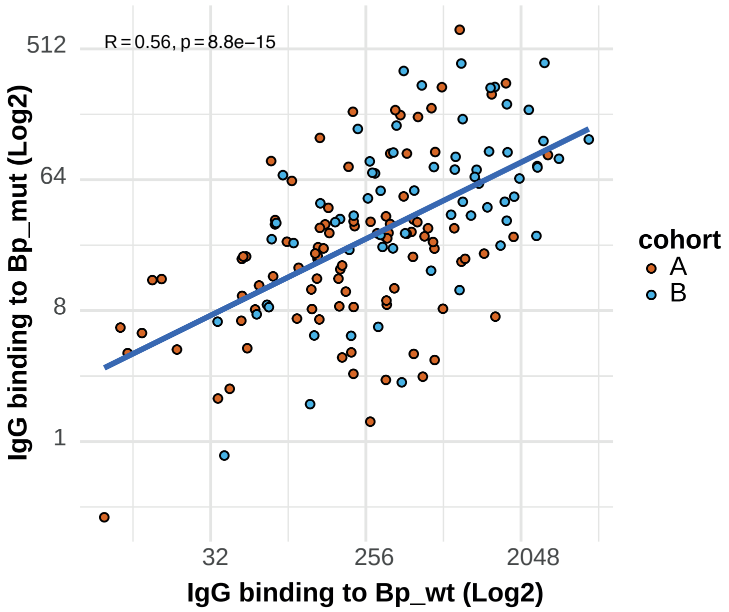

R = 0.56, p = 8.8e−15
512
64
8
1
32
256
2048
IgG binding to Bp_mut (Log2)
IgG binding to Bp_wt (Log2)
cohort
A
B

Figure S9. Antibody deposition correlation between *Bp_mut* and *Bp_wt*. A spearman correlation was performed on the Log2-transformed mean fluorescence intensity (MFI) IgG binding to *Bp_mut* with *Bp_wt* mucosal IgG deposition levels (log2-transformed mean fluorescence intensity) at baseline in the aP-primed cohorts. Data are N = 32 individuals for cohort A and N= 22 individuals for cohort B. A two-sided spearman correlation was performed and the correlation factor R and p-value are depicted in the figure.

Table S1. List of bacterial strains used in this study. Bacterial strain name, genotype description and reference are indicated.

**Strain**	**Genotype and relevant description**	**Reference**
***Escherichia coli* strains used for pSS4245 derivative construction and conjugation**		
XL1-Blue	*rec*A1 *end*A1 *gyr*A96 *thi*-1 *hsd*R17 *sup*E44 *rel*A1 *lac*<br>F´ *pro*AB *lac*IqZΔM15 Tn10 Tetr	Stratagene
SM10 λ*pir*	*thi thr leu ton*A *lac*Y *sup*E *rec*A::RP4-2- Tc::Mu Km λ*pir*	Simon et al., 1983
***Bordetella pertussis* strains**		
Bp wt	*Bp* B1917*;* wild type *Bordetella pertussis* B1917*; fim2-1, fim3-2, ptx*P3*, ptx*A1*, ptx*B2*, ptx*C2*, ptx*D1*, ptx*E1*, prn*2	Bart et al., 2014
*Bp*_mut (Δ*fhaB*, Δ*prn*, Δ*ptxS1-S3*)	Triple deletion mutant of *Bp* B1917, carrying an in-frame deletion of codons N4 - T3588 of the *fhaB* gene, an in-frame deletion of codons N2 - W910 of the *prn* gene and an in-frame deletion of codons R2 of *ptxS1* to C227 of *ptxS3*, thus deleting the *ptx* operon.	this study

Table S2. List of PCR primers used for construction of *B. pertussis* mutant strains. The mutant strain and primer sequence (5´- 3´) are provided.

**Mutant strain / mutation**	**Primer sequence for / rev**
B1917 Δ*fhaB*	FhaNotI_for CTGCGGCCGCGGCATTGATGACCTCGTGCAG<br>FhaSpeI_rev GAACTAGTCGTGTTCATATTCCGACCAGC<br>FhaSpeI_for CTACTAGTAACAAATAGGTAGTCGCGGCCTG<br>FhaBamHI_rev GAGGATCCCATGCCGCCTTGCCGCTTTAC
B1917 Δ*prn*	Prn NotI_for CTGCGGCCGCAGCAAGACTGCGAGCTGCTG<br>Prn SpeI_rev CTACTAGTCATTGGATGCCAGGTGGAGAG<br>Prn SpeI_for CTACTAGTTAAAGCGAGGAGGGTCTATCC<br>Prn BamHI_rev CTGGATCCTGGTCAGCGAGGGCGTGC
B1917 Δ*ptxS1-S3*	Ptx delta SpeI_for CTACTAGTGCGGTGCTGGAACATATCCG<br>Ptx delta SacI_rev CTGAGCTCCATCCCGTCTTCCCCTCTG<br>Ptx delta SacI_for CTGAGCTCTGAGCCGCCGGCTCGGATC<br>Ptx delta BamHI_rev CTGGATCCCAGCGGCGCATAGACGGTAC
