# Supplementary material for: Prior exposure to B. pertussis shapes the mucosal antibody response to acellular pertussis booster vaccination
Source: Nat Commun. 2022 Dec 2;13:7429. doi: 10.1038/s41467-022-35165-w (PMC9716536; doi:10.1038/s41467-022-35165-w)
Supplement: Supplementary file 3 — Reporting Summary [file 41467_2022_35165_MOESM3_ESM.pdf]

nature portfolio

Corresponding author(s): Dimitri Diavatopoulos

Last updated by author(s): Nov 11, 2022

Reporting Summary

Nature Portfolio wishes to improve the reproducibility of the work that we publish. This form provides structure for consistency and transparency in reporting. For further information on Nature Portfolio policies, see our Editorial Policies and the Editorial Policy Checklist.

Statistics

For all statistical analyses, confirm that the following items are present in the figure legend, table legend, main text, or Methods section.

n/a	Confirmed	
☐	☒	The exact sample size (*n*) for each experimental group/condition, given as a discrete number and unit of measurement
☐	☒	A statement on whether measurements were taken from distinct samples or whether the same sample was measured repeatedly
☐	☒	The statistical test(s) used AND whether they are one- or two-sided<br>*Only common tests should be described solely by name; describe more complex techniques in the Methods section.*
☒	☐	A description of all covariates tested
☐	☒	A description of any assumptions or corrections, such as tests of normality and adjustment for multiple comparisons
☐	☒	A full description of the statistical parameters including central tendency (e.g. means) or other basic estimates (e.g. regression coefficient) AND variation (e.g. standard deviation) or associated estimates of uncertainty (e.g. confidence intervals)
☐	☒	For null hypothesis testing, the test statistic (e.g. *F*, *t*, *r*) with confidence intervals, effect sizes, degrees of freedom and *P* value noted<br>*Give P values as exact values whenever suitable.*
☒	☐	For Bayesian analysis, information on the choice of priors and Markov chain Monte Carlo settings
☒	☐	For hierarchical and complex designs, identification of the appropriate level for tests and full reporting of outcomes
☐	☒	Estimates of effect sizes (e.g. Cohen's *d*, Pearson's *r*), indicating how they were calculated

Our web collection on statistics for biologists contains articles on many of the points above.

Software and code

Policy information about availability of computer code

Data collection	FACS LSR-II (BD biosciences, SanJose, CA, USA) for antibody deposition and Bio-Plex LX200 for antibody concentrations.
Data analysis	Analysis of antibody deposition was performed with FlowJo Version X (FlowJo, LLC, Ashland, OR, USA). Analysis of antibody concentrations was performed with Bio-Plex Manager 6.2 (Bio-Rad Laboratories, Hercules, CA). All statistical analyses were performed using the programming language “R” version 4.1.3 in the Rstudio environment version 2022.02.1 with libraries 'ggpubr' and ‘tidyverse’ used for data cleaning and ‘ggplot2’ used for plotting.

For manuscripts utilizing custom algorithms or software that are central to the research but not yet described in published literature, software must be made available to editors and reviewers. We strongly encourage code deposition in a community repository (e.g. GitHub). See the Nature Portfolio guidelines for submitting code & software for further information.

Data

Policy information about availability of data

All manuscripts must include a data availability statement. This statement should provide the following information, where applicable:

- Accession codes, unique identifiers, or web links for publicly available datasets
- A description of any restrictions on data availability
- For clinical datasets or third party data, please ensure that the statement adheres to our policy

The processed data generated in this study are provided in the Source Data file. The raw data are available from the corresponding author upon reasonable request. The raw data are not publicly available due to data and volunteer’s privacy laws. Source Data are provided with this paper.

Field-specific reporting

Please select the one below that is the best fit for your research. If you are not sure, read the appropriate sections before making your selection.

☒ Life sciences ☐ Behavioural & social sciences ☐ Ecological, evolutionary & environmental sciences

For a reference copy of the document with all sections, see nature.com/documents/nr-reporting-summary-flat.pdf

Life sciences study design

All studies must disclose on these points even when the disclosure is negative.

Sample size

The Dutch part of the BERT Tdap-IPV booster study aimed to include 36 children aged 7-10 years, 36 adolescents aged 11-15 years, 25 young adults aged 20-34 years and 25 older adults aged 60-70 years. It was estimated that a sample size of 108 per child cohort across all countries (the Netherlands, Finland, and the United Kingdom) was sufficient to give 80% power to detect a standardised difference in log-anti-PT-IgG at one month post booster of 0.42 IU/mL between age cohorts, allowing 15% loss to follow-up or sample loss. In the end, 36 children were included, 48 adolescents (in order to have an equal distribution between aP and wP primed adolescents), 25 young adults, and 25 older adults. For the immunological sub-analysis presented in this manuscript, we focused on participants who were vaccinated with aP-vaccines during infancy, with a sample size of N = 32 for children between 7-10 years and N = 22 for adolescents between 11-15 years, due to missing data and no consent after inclusion in the study.
For the controlled human infection model, 54 subjects were screened. The dose of the inoculum, starting at 1000 colony-forming units, was adjusted after each fifth subject to achieve colonisation of 70% of the subjects, with a minimum of 10 colonized volunteers in the final inoculation dose. Subjects were excluded based on high anti-PT IgG titers and other criteria. In the end, 34 subjects were challenged with different doses.

Data exclusions

For the booster vaccination study, we measured antibody deposition and antibody concentrations for 32 children and 22 adolescents. Samples were excluded based on missing data and no consent for mucosal sampling.
For the controlled human infection model, we only measured antibody deposition for the 15 volunteers who received an inoculum dose of 10^5 CFU, which was identified as the standard inoculum dose required to reach at least 70% colonisation.

Replication

Antibody deposition was measured once, after reproducibility of the data was confirmed by replication of a subset of samples (in which correlation was high). MIA analyses were performed in independent duplicates, which were measured on seperate days and of which the %CV is calculated. Sample replication was successful when %CV was below 47. Both assays included QC samples and a reference curve, to enable batch effect detection, which showed high assay stability.

Randomization

No randomization was performed between cohort A and B, as the aim of the study was to compare immunogenicy of Tdap-IPV in different age groups, so randomization by age was not possible. Demographical data of study cohorts is shown in Table 1. Information on primary pertussis vaccination background (i.e. number of doses and vaccine formulation) was accounted for in subanalyses and did not influence the observed differences between the cohorts.
The controlled human infection study was a dose escalation study, not an intervention study, and therefore does not include a randomization step.

Blinding

The BERT study was an open label study and therefore not blinded to study participants or clinical staff, as the overall objective was to compare the immune response to the same intervention (Tdap-IPV) in different age groups.
Similarly, the pertussis controlled human infection model was also not blinded to study participants or clinical staff. The samples that we used in this manuscript were all obtained from volunteers who received the same inoculum dose, i.e. 10^5 colony forming units of B. pertussis B1917 (standard inoculum, see manuscript).

All sample measurements (Ab deposition assay, MIA) were performed in a blinded manner. Information on the subject ID group allocation were stored in a separate location and were not accessible to the team doing the lab measurements. The group allocation was only used for data analysis after the assay results for each sample were validated and passed QC.

Reporting for specific materials, systems and methods

We require information from authors about some types of materials, experimental systems and methods used in many studies. Here, indicate whether each material, system or method listed is relevant to your study. If you are not sure if a list item applies to your research, read the appropriate section before selecting a response.

Materials & experimental systems

n/a	Involved in the study
☐	☒ Antibodies
☒	☐ Eukaryotic cell lines
☒	☐ Palaeontology and archaeology
☒	☐ Animals and other organisms
☐	☒ Human research participants
☐	☒ Clinical data
☒	☐ Dual use research of concern

Methods

n/a	Involved in the study
☒	☐ ChIP-seq
☐	☒ Flow cytometry
☒	☐ MRI-based neuroimaging

Antibodies

Antibodies used

For confirming the absence of protein products in Bordetella pertussis B1917, monoclonal antibodies for Bp Ptx toxin subunit S1 (63.1 G9), SC-57639, Lot n. J2720, Santa Cruz Biotechnology, dilution 1:500 MAb recognizing the N-terminal region of Fha, clone F1, (kind gift of C. Locht, Institut Pasteur Lille, France, dilution 1:100) and polyclonal rabbit serum raised against the Prn (home made, dilution 1:10,000) were used.

For detection of human IgM, IgA and IgG binding to bacteria using flow cytometry, secondary antibodies were used. These were goat polyclonal anti-human IgM-AF647 (Fc-specific, Jackson ImmunoResearch), goat polyclonal anti-human IgG-PE (Fc-specific, Jackson ImmunoResearch), and goat polyclonal anti-human IgA-FITC (α-specific, Sigma-Aldrich) were used in a dilution of 1:500 for IgM and IgG and 1:100 for IgA.

For detection of PRN, PT, FHA and FIM-specific IgG and IgA in the multiplex immunoassay (MIA), goat anti-human IgG-PE (Fc-specific, Jackson ImmunoResearch), and goat anti-human IgA-PE (Fc-specific, SouthernBiotech) were used in a dilution of 1:200.

Validation

Validation of the monoclonal antibodies is done by using purified proteins as positive controls and is described in the following manuscripts: See references for more information and validation: doi: 10.1016/j.ijmm.2015.11.003; doi: 10.3390/ijms232012598. For detection using flow cytometry: doi: 10.1186/s12967-019-1928-x. For detection in the MIA doi: 10.1016/j.jim.2008.02.018.

Human research participants

Policy information about studies involving human research participants

Population characteristics

In the booster vaccination study, cohort A had a mean age of 8.5 (95% CI: 8.4-8.6) and cohort B had a mean age of 13.6 (95% CI: 13.2-13.9). Of cohort A, 50% was female, of cohort B, 35.4% was female.
In the controlled human infection model, the median age of the 34 inoculated subjects was 26 (IQR: 21-35), with 41% females. The median weight was 74 kg (IQR: 65-83) and median height 176 cm (IQR:170-183).

Recruitment

Participants of the booster vaccination study were recruited by mail-outs to a sex balanced sample in the region of Hoofddorp, attained via the Municipal Administration (adolescents, young adults, and older adults) or through the NIP (children). Self-selection bias may be possible as participants who were interested in the study may differ from the general population. However, it is unlikely that this will influence differences between the study cohorts as they were recruited in the same manner and we expect any potential bias to be random.
Participants of the controlled human infection model were recruited via an online advertisement.

Ethics oversight

The booster vaccination study was approved by the Medical Research Ethics Committees United in the Netherlands (MEC-U, NL60807.100.17-R17.039).
The controlled human infection model was approved by the Oxford A Research Ethical Committee in the UK (NCT03751514, reference 17/SC/0006).

Note that full information on the approval of the study protocol must also be provided in the manuscript.

Clinical data

Policy information about clinical studies

All manuscripts should comply with the ICMJE guidelines for publication of clinical research and a completed CONSORT checklist must be included with all submissions.

Clinical trial registration

The booster vaccination study was registered at the EU Clinical Trial database (EudraCT number 2016-003678-42).
The controlled human infection model was registered with ClinicalTrials.gov (NCT03751514).

Study protocol

The protocol for the controlled human infection study has been published, see: doi: 10.1136/bmjopen-2017-018594. The full protocol for the BERT-study can be sent upon reasonable request to the corresponding author. For the brief protocol, see ClinicalTrials.gov.

Data collection

The booster vaccination study was conducted by the Spaarne Academy (Spaarne Hospital, Hoofddorp, the Netherlands). Mucosal lining fluid (MLF) samples were collected at day 0, day 28 (± 4 days), and 1 year (± 4 weeks) post-vaccination and subsequently stored at -80°C until elution. After elution of the MLF samples, the eluate was placed into aliquots and tranferred to -80°C until further analyses. Antibody deposition data was generated using FACS LSR-II (BD biosciences, SanJose, CA, USA) and antibody concentrations

were generated using Bio-Plex LX200.
Volunteers of the controlled human infection model were admitted to the National Institute for Health Research (NIHR)-Clinical research facility (CRF) at Southampton for 17 days. Mucosal lining fluid (MLF) samples were collected 7 days before challenge and 28 days after challenge and subsequently stored at at -80°C until elution. After elution of the MLF samples, the eluate was placed into aliquots and tranferred to -80°C until further analyses. Antibody deposition data was generated using FACS LSR-II (BD biosciences, SanJose, CA, USA) and antibody concentrations were generated using Bio-Plex LX200.

Outcomes

The primary outcome measure of the BERT study is:
- change from baseline in pertussis toxin-specific IgG antibody levels in serum to 28 days after vaccination with Tdap-IPV (doi: 10.1016/j.ebiom.2021.103247).

Secondary outcome measures for the BERT study include:
- concentrations of pertussis toxin (PT) specific IgG antibody one year after vaccination with Tdap-IPV (this manuscript)
- change from baseline of antigen-specific IgG antibody levels against other pertussis vaccine antigens (FHA, PRN, FIM2/3) to 28 days and 1 year after vaccination with Tdap-IPV (this manuscript)
- change from baseline of functional pertussis-specific antibody levels (e.g. bacterial antibody binding) 28 days and 1 year after vaccination with Tdap-IPV (this manuscript)
- change from baseline of B cell responses against Bordetella pertussis vaccine proteins after vaccination with Tdap-IPV (doi: 10.3389/fimmu.2022.864674)

The clinical protocol (doi: 10.1136/bmjopen-2017-018594) for the dose-escalation phase of the controlled human B. pertussis infection study has been described elsewhere.

The primary outcome measure for the dose-escalation phase of the controlled human infection study was:
- the inoculum dose required to cause the safe colonisation of 70% of volunteers who are challenged (doi: 10.1093/cid/ciz840)

Secondary outcome measures:
- Immune Responses to exposure to Bordetella pertussis (e.g. bacterial antibody deposition, this study)

In this manuscript, the first three secondary research questions of the BERT study have been discussed by analyzing the levels of bacterial antibody binding in mucosal lining fluid before and after booster vaccination using the antibody deposition assay, and the concentration of antigen specific mucosal antibodies at various timepoints using the MIA.

Flow Cytometry

Plots

Confirm that:

☒ The axis labels state the marker and fluorochrome used (e.g. CD4-FITC).

☒ The axis scales are clearly visible. Include numbers along axes only for bottom left plot of group (a 'group' is an analysis of identical markers).

☒ All plots are contour plots with outliers or pseudocolor plots.

☒ A numerical value for number of cells or percentage (with statistics) is provided.

Methodology

Sample preparation

To measure antibody deposition on the bacterial strains, 2 x 10^6 colony forming units (CFU) of bacteria were incubated with 50% heat-inactivated (30 minutes at 56°C; to inactivate complement and other inhibitory components) mucosal lining fluid samples in PBS + 2% BSA for 30 minutes at 37°C + 5% CO2 while shaking. Subsequently, the bacteria-antibody complexes were centrifuged and fixed in 2% paraformaldehyde for 20 minutes at room temperature. Bacteria were then centrifuged again and resuspended in PBS + 2% BSA containing anti-human IgM-AF647, anti-human IgG-PE, and anti-human IgA-FITC. After 15 minutes incubation at room temperature, surface-bound IgM, IgG, and IgA was measured by a 'Euroflow' standardized flow cytometry.

Instrument

FACS LSR-II (BD biosciences, SanJose, CA, USA).

Software

Data were analyzed using FlowJo Version X (FlowJo, LLC, Ashland, OR, USA) and R.

Cell population abundance

To measure the same amount of bacteria in each experiment, we set a stopping gate of 10,000 bacteria on the flow cytometer, gated on FSC/SSC.

Gating strategy

First, we gated bacteria on FSC/SSC based on the positive control sample included. We then used this gate for the mucosal lining fluid samples and checked if all bacterial populations fell within this gate. Of this gated population, we determined the mean fluorescence intenstiy of IgM, IgG, and IgA.

☒ Tick this box to confirm that a figure exemplifying the gating strategy is provided in the Supplementary Information.
